# The abTEM code: transmission electron microscopy from first principles

**DOI:** 10.12688/openreseurope.13015.2

**Published:** 2021-05-21

**Authors:** Jacob Madsen, Toma Susi

**Affiliations:** 1 Faculty of Physics, University of Vienna, Vienna, 1090, Austria

**Keywords:** transmission electron microscopy, electron scattering, image simulation, density functional theory, molecular dynamics, open source, Python

## Abstract

Simulation of transmission electron microscopy (TEM) images or diffraction patterns is often required to interpret experimental data. Since nuclear cores dominate electron scattering, the scattering potential is typically described using the independent atom model, which completely neglects valence bonding and its effect on the transmitting electrons. As instrumentation has advanced, new measurements have revealed subtle details of the scattering potential that were previously not accessible to experiment.

We have created an open-source simulation code designed to meet these demands by integrating the ability to calculate the potential via density functional theory (DFT) with a flexible modular software design. abTEM can simulate most standard imaging modes and incorporates the latest algorithmic developments. The development of new techniques requires a program that is accessible to domain experts without extensive programming experience. abTEM is written purely in Python and designed for easy modification and extension.

The effective use of modern open-source libraries makes the performance of abTEM highly competitive with existing optimized codes on both CPUs and GPUs and allows us to leverage an extensive ecosystem of libraries, such as the Atomic Simulation Environment and the DFT code GPAW. abTEM is designed to work in an interactive Python notebook, creating a seamless and reproducible workflow from defining an atomic structure, calculating molecular dynamics (MD) and electrostatic potentials, to the analysis of results, all in a single, easy-to-read document.

This article provides ongoing documentation of abTEM development. In this first version, we show use cases for hexagonal boron nitride, where valence bonding can be detected, a 4D-STEM simulation of molybdenum disulfide including ptychographic phase reconstruction, a comparison of MD and frozen phonon modeling for convergent-beam electron diffraction of a 2.6-million-atom silicon system, and a performance comparison of our fast implementation of the PRISM algorithm for a decahedral 20000-atom gold nanoparticle.

## 1 Introduction

Transmission electron microscopy (TEM) is one of the most versatile and powerful experimental tools for the imaging and diffraction of structures ranging from the micrometer scale with sub-Ångström resolution now routinely achievable in modern aberration corrected instruments. In TEM, information about the sample structure is encoded in the scattering of the electron waves by the full electromagnetic potential of the specimen, which is dominated by the atomic electrostatic potentials. These potentials include the contribution of the screened nuclear cores as well as the valence electron density of the sample, and since valence electrons are responsible for binding the material together, studying them is of significant scientific interest.

The modern electron microscope should be an ideal tool for the high-resolution imaging of charge redistribution caused by chemical bonding, but these measurements are a challenge because only a small fraction of the total electrons in a material participate in bonding, and because the dense cores dominate the scattering signal. However, as improvements in instrumentation and techniques continue rapidly, this is likely to increasingly change, as evidenced by the surging popularity of techniques such as four-dimensional scanning transmission electron microscopy (4D-STEM) combined with ptychography in materials science, and cryogenic microcrystal electron diffraction in structural biology.

To reliably quantify subtle differences in the scattering signal, precise alignment of the instrument and a careful comparison between theoretical models and experiments are required. The use of image simulations has long aided this process, and many excellent codes have been developed. However, these have exclusively relied on the independent atom model (IAM), which approximates the specimen potential as a superposition of isolated atoms, completely neglecting chemical bonding. A growing number of studies are going beyond the IAM by calculating the potential using density functional theory (DFT) ^1– 7^. As expected, these studies find a better agreement for a range of different materials when comparing to measurements that are sensitive to valence electron density, such as holography and various forms of phase-contrast imaging.

The most common image simulation method is the multislice algorithm, and there is no shortage of codes implementing it 8– 19, though the degree of support and documentation varies widely. Implementations have remained largely similar for a good while, apart from some iterative improvements. One significant recent development has been to use one (or more) graphics processing units (GPUs) for accelerating the calculations. In terms of methods, the most important recent advancement is the development of the PRISM algorithm, which massively accelerates scanning TEM (STEM) simulations ^20^.

Here, we present the abTEM code, which is a new multislice image simulation package created to seamlessly merge DFT and other atomistic modeling methods with electron scattering simulations, providing a much easier way of performing TEM simulations with an *ab initio* description of bonding. We have implemented both the multislice and PRISM algorithms to simulate all the standard imaging modes.

abTEM is further distinguished from existing codes by its pure Python implementation and focus on user extendability. We show that thanks to the effective utilization of open-source libraries, the performance of abTEM is as good or even better compared to similar codes implemented in programming languages that have traditionally been considered superior in performance, such as C or Fortran. abTEM already includes fast GPU implementations of all of its algorithms on widely used NVidia GPUs, and support for products from AMD is planned for the near future.

abTEM is an open-source project under the GPLv3 license, and we welcome contributions via our open Github repository ^21^. Documentation and code examples are found online. The code was announced at the M&M 2020 meeting ^22^, and it supersedes and replaces our earlier P yQSTEM code ^6^.

This article is organized as follows: in Section 2, we discuss the physical methodology behind abTEM, including its implementation of the multislice electron scattering algorithm, IAM and *ab initio* potentials, the PRISM algorithm, and inelastic and thermal diffuse scattering. Next, in Section 3 we give brief remarks on our implementation details, followed by illustrative code examples. In Section 4, we discuss in turn programmatic modularity, dependencies and availability, interactive dashboards, and finally GPU memory management. We then turn to actual use cases in Section 5, providing novel results on systems that highlight how easy it is to use *ab initio* electrostatic potentials and how fast and efficient abTEM is. Finally, we end with a brief conclusion in Section 6, including features that we plan to develop next.

## 2 Methods and algorithms

### 2.1 Multislice

The multislice algorithm can be used to simulate any kind of TEM measurement: what differs is only how the input wave function of the electron probe is defined and how the scattered exit wave function is detected. Since the multislice algorithm is conceptually quite simple and discussed in detail elsewhere ^8^, we provide only a brief sketch of the method here.

In the multislice algorithm, the specimen electrostatic potential is divided into thin slices along the beam propagation direction (which by abTEM convention is the *z* axis). Scattering is calculated by alternating so-called phase object transmission through each slice with propagation of the wave to the next. For an electron wave *_n_* impinging on slice number *n*, this can be expressed as

$${}_{\left(n+1\right)}=p*({}_{n}t_{n}),$$

where *p* is the Fresnel free-space propagator, * represents the convolution operation, and the transmission function *t _n_* is defined as

$$t_{n}(x,y)=\exp\;(i\sigma V_{n}(x,y)),$$

where *i* is the imaginary unit, *σ* is the interaction constant dependent on the electron wavelength λ, and

$$\;V_{n}(x,y)=\int_{z_{n}}^{z_{n}+\text{Δ}z}V(x,y,z)dz\;(1)$$

is the *n*’th projected potential slice with thickness ∆ *z*.

The convolution is conveniently calculated as a multiplication in Fourier space via a succession of a forward and an inverse Fourier transform. Another Fourier transform is needed to bandwidth-limit the transmission function and the Fresnel propagator to 2 */*3 of the Nyquist frequency to avoid aliasing artifacts ^8^.

The Fourier transforms are carried out efficiently using the fast Fourier transform algorithm implemented in the efficient FFTW ^23^ and cuFFT libraries on CPU and CUDA-enabled GPUs, respectively.

### 2.2 Potentials

The electrostatic potential of a specimen determines how transmitting electrons scatter and thus connects the properties of the material to the resulting images or diffraction patterns. Conversely, by directly analyzing scattered intensities or comparing them to simulations of the specimen electrostatic potential, the properties of the sample can be deduced. Fundamentally, the electrostatic potential is directly derived from the electron density of the atoms in a specimen, which is described by their quantum mechanical many-body wave function.

Since this cannot be analytically solved except for the very simplest of molecules, various approximations have been developed, such as early Hartree-Fock-Slater and Dirac-Fock formalisms. In the context of electron scattering, these highly accurate but computationally extremely expensive techniques can be used to parametrize (IAMs). However, there is increasing interest in a fully *ab initio* approach to calculating the specimen potential ^22^. The most widely used and powerful such method is DFT, where the many-body problem of *N* electrons with 3 *N* spatial coordinates is reduced to a variational solution for the three spatial coordinates of the electron density.

While abTEM is designed explicitly for *ab initio* potentials, IAM potentials can in many cases be sufficient or useful: for example, typical annular dark-field contrast in STEM is dominated by nuclear scattering and thus well described by the IAM, and in general, comparing images or diffraction patterns simulated with *ab initio* potentials to the IAM allows the effects of chemical bonding to be elucidated. Thus, abTEM supports common parametrized IAM potentials in addition to its integration with DFT methods.

### 2.3 Parametrized potentials

A potential parametrization, as pioneered by Doyle and Turner ^24^, is a numerical fit to atomic electron scattering factors calculated from first principles, describing the radial dependence of the potential for each element. abTEM supports two of the most accurate recent parametrizations: that by Lobato and Van Dyck ^25^ (which is the abTEM default) and by Kirkland ^8^.

Building upon earlier work Weickenmeier and Kohl ^26^ as well as Peng *et al.* ^27^, Kirkland accurately fitted a combination of Gaussians and Lorentzians to Dirac-Fock scattering factors, providing a widely used and robust parametrization that was distributed digitally from 1998 onwards. In 2014, Lobato and Van Dyck improved the quality of the fit further, using hydrogen’s analytical non-relativistic electron scattering factors as basis functions to enable the correct inclusion of all physical constraints, providing to date the most accurate universal neutral atom parameter set ^25^. For a comparison of several parametrizations to each other as well as quantitative experimental data for 2D materials, please see Ref. 6.

***2.3.1 ab initio potentials.*** Although the ground-state electron density for all electrons could be numerically solved in DFT, the description of electron wave functions near the nuclei is computationally very expensive, and thus most practical approaches have adopted some partition between the core and valence regions. While reference methods such as linearized full-potential augmented plane waves (FLAPW) are very accurate, their computational expense limits them to around a hundred atoms, which is insufficient for most TEM simulations.

In recent years, pseudopotential ^28^ and projector-augmented wave (PAW) methods ^29^ have offered much greater computational efficiency. In both, the core electrons are not described explicitly but replaced by a smooth pseudo-density in the former, and in the latter by smooth analytical projector functions in the core region. The PAW method is arguably better suited for obtaining efficient and accurate *ab initio* all-electron electrostatic potentials, since inverting the projector functions allows the true core electron density to be analytically recovered (for an extended discussion, see Ref. 22). As such, this is the approach chosen for abTEM, specifically via the grid-based DFT code GPAW.

In the PAW formalism ^29^, the total charge density *ρ*( **r**) is a sum of the squared explicitly computed all-electron valence wave functions, the (frozen) core electron density derived from the PAW projector functions, and the nuclei that are treated as point charges. The charge density is divided into a smooth part $\tilde{\rho }$ ( **r**) plus corrections for each atom *a*: *ρ ^a^* ( **r**) − ${\tilde{\rho }}^{a}$ ( **r**), where the smooth part is given as pseudo wave functions and pseudo core charges. We can obtain the electrostatic potential *v*( **r**) by solving the Poisson equation in two separated steps for the pseudo part and within the atomic augmentation spheres, and finally adding smeared nuclear charges to avoid the corrections diverging as *−Z ^a^/r* near the nuclei in the total charge density.

The wavefunctions, electron density, and potential are described on real-space numerical grids, whose density controls the accuracy of the calculation (though not in a variational manner, unlike in traditional plane-wave bases also supported by GPAW). A detailed description of the code is given in Refs. 30, 31, while the all-electron electrostatic potentials derived from it are described in our earlier work in Ref. 6.

It is worth noting that like other codes, abTEM currently uses only the electro *static* potential, whereas electrons in truth interact with both electric and magnetic fields via the Lorentz force. This approximation is well justified by the fact that magnetic interactions are much weaker, but there is emerging interest in multislice simulations that also account for magnetism ^32^. An *ab initio* approach is obviously ideally suited for such modeling, and our integration with the highly scalable GPAW code makes large magnetic domains or nanostructures feasible to treat from first principles.

***2.3.2 Potential slicing.*** The multislice method requires a mathematical slicing of the potential into *xy* planes as given by Equation 1. abTEM implements two alternative methods for projecting the potential: the formally correct finite numerical integrals, and a less accurate but commonly used and much faster method of using analytically solvable infinite integrals.

The finite projection method is based on evaluating the singular potential functions in real space. The singularities are removed by effectively convolving the 3D potential with a Gaussian whose standard deviation is equal to the real-space grid spacing. This is implemented using the convolution theorem; the 3D scattering factor is multiplied by a Gaussian, and the resulting radial function is Fourier-transformed to obtain the a radial potential function without a singularity.

We evaluate the the finite numerical projection integrals in Equation 1 following the work of Lobato and Van Dyck ^13^. The integrals are handled by the double exponential Tanh–Sinh quadrature ^33^, which is designed for accurate evaluation of functions with endpoint singularities. Integrals across the sharply peaked cores are split into two integrals at the atomic position and the number of weights and nodes of the quadrature are determined such that the error is smaller than a given tolerance using a worst-case error estimate.

Instead of performing the expensive integral at each grid point, it is calculated along a radial line and interpolated on the simulation grid. Due to the rapid change of the atomic potentials near the core, the evaluation points of the integrals are geometrically spaced. The potential is set to zero outside a cutoff radius, determined for each atomic species such the error is smaller than a given tolerance. The use of the cut-off radius creates a discontinuity, hence we use a tapering cut-off function.

For simulations with thousands of atoms, a large proportion of the integrals are likely to be almost identical, and thus a cache is automatically used for saving the integrals and reusing their results if integration limits are identical within a given tolerance.

The potential slices are calculated in parallel using multithreading on both GPUs and CPUs. If the entire potential is too large to hold in memory, it is possible to calculate a smaller number of slices as they are needed. The maximum number of slices calculated in parallel is automatically estimated based on available system memory.

Most other codes use only the infinite projection method for calculating the projected potential, which assigns the infinite projection of each atom to a single slice. The fastest method of implementing this is by placing delta functions at the atomic positions and convolving the superposition with analytical potentials ^11^. The convolution is efficiently evaluated by multiplying the Fourier-transformed superposition of delta functions with the 2D scattering factors, thereby also avoiding the issue of real-space singularities. The result is divided by a Sinc function to compensate for the finite size of the discretized delta functions.

This infinite projection method is up to 100 times faster than using finite projection integrals. The error can be up to a few percent in regions between the atoms, but much less when nuclear scattering is dominant. An example in our online repository explores these differences ^21^.

### 2.4 PRISM

Although it is universally applicable and conceptually simple, the multislice algorithm is not very efficient for large-scale STEM simulations, where the electron probe scans across the specimen: while the atomic potentials can be reused for different probe positions, the remainder of the calculation must be run independently. For STEM simulations consisting of thousands or even millions of probe positions, this is very costly. This problem is directly addressed by the recently developed PRISM algorithm ^16, 20^.

The PRISM algorithm takes advantage of the fact that the electron probe can be expressed as a plane-wave expansion. Using the linearity of the multislice algorithm with respect to the wave function, the individual plane-wave basis functions can be propagated through the specimen independently. The set of plane waves at the exit surface, defined as the scattering matrix, can be coherently summed to calculate a scattered probe. The position and aberrations of the scattered probe can be chosen by multiplying each plane wave, *ϕ _nm_*, by an appropriate complex coefficient prior to summation:

$$(r,r_{0})=\sum_{nm}\alpha_{nm}(r_{0})\phi_{nm}(r),\;\phi_{nm}\;=\exp(-2\pi iq_{nm}\;\cdot \;r),\;(2)$$

where the coefficient, ***α*** *_nm_*, depends on the aberrations and the desired probe position *r*_0_. The wave vector, *q _nm_*, must fulfill

$$q_{nm}\;=\;(nf\Delta_{q},\;mf\Delta_{q}),\;(3)$$

where Δ *_q_* is the Fourier space pixel size, the integer *f* is the interpolation factor, and the integers *m* and *n* represents the plane-wave index fulfilling

$$\;\sqrt{m^{2}+n^{2}}f\lambda \text{Δ}q\;\leq \;\alpha_{max},\;(4)$$

where *α _max_* is the probe convergence semi-angle. The size of the basis is naturally limited by the aperture and can be further reduced by a factor of *f* ^2^ at the cost of accuracy. Increasing the interpolation factor, and thus keeping only every *f*'th coefficient in Fourier space, corresponds to tiling the probe *f* times in real space, hence the interpolation factor should be chosen such that the tiled probes does not interact. To aid users abTEM implements methods for estimating the error and choosing an appropriate level of interpolation.

When the interpolation factor is greater than one, the result needs to be cropped so only the desired probe and not its repetitions are included. Due to the memory-size of the scattering matrix the computational cost of the cropping operation is non-trivial. In the original implementation ^16^, the scattering matrix is cropped before the sum reduction in Equation 2 is performed, ensuring that a minimum number of operations are necessary. Our algorithm differs by cropping after performing the reduction for a batch of positions: a chunk of the scattering matrix, large enough for calculating all positions in the batch, is selected and if necessary, is transferred to the GPU. The coefficients for the entire batch are calculated, and the reduction is performed simultaneously for all positions in the batch using multithreaded parallelization. Lastly, the cropping is performed on the much smaller reduced array.

Our implementation requires more floating-point operations than strictly necessary, but calculations are performed with fewer memory transfers and expensive kernel launches from Python. To minimize excess work, probe-position batches are taken as compact squares in order to have as much overlap of the necessary parts of the scattering matrix as possible. This implementation has the unusual feature that its speed does not necessarily increase monotonically with the batch size: The amount of excess work increases with the batch size, so once full thread utilization is reached, further increase of the batch size may degrade the performance.

In Section 5, we directly compare the abTEM implementation of PRISM to the Prismatic code ^16^, demonstrating the highly competitive performance of our pure Python code.

### 2.5 Inelastic scattering

The basic version of the multislice algorithm assumes that the electron beam scatters only elastically. In real materials, the electrons also undergo inelastic energy loss, which affects their interference and contributes to backgrounds. Furthermore, the atoms of the target are not static but in constant motion due to zero-point vibrations and thermal phonon occupations.

Inelastic scattering can be approximately modeled using an absorptive potential ^34^, where the imaginary part of a complex electrostatic potential is used to describe the loss of electrons from the elastic channel. However, in this approach, the inelastically interacting electrons are simply removed from an otherwise purely elastic scattering calculation. Although this is computationally efficient, the method’s serious weaknesses are that the electron flux is not conserved, and high-angle scattering is underestimated. For the case of phonon scattering, this limitation is overcome in the widely used frozen phonon model (see below).

The frozen phonon model has become standard in most multislice programs. However, there is also increasing interest in explicitly simulating spectra for low energy losses, which can be theoretically quite challenging ^35, 36^. Very recently, Zeiger and Rusz developed a novel method for modeling low-loss electron energy-loss spectroscopy (EELS) based on molecular dynamics and multislice simulations ^37^, for which our code is ideally suited. Moving up in energy, plasmon scattering is, to our knowledge, not included in any publicly available code, perhaps because they are less important for high-angle scattering ^38^. Plasmon modeling following a Monte Carlo method by Mendis ^39^ is planned for a future release.

Finally, inner-shell ionization is of particular interest as a spectroscopic fingerprint of elements and their bonding. EELS, including dynamical scattering, can be modeled by combining multislice simulations with the transition potentials for the elements of interest ^12^. Simulating EEL spectrum images used to be extremely computationally demanding, but recently a much faster method was developed by Brown ^40^. These algorithms are currently being implemented into abTEM, further improving its *ab initio* capabilities.

***2.5.1 Thermal diffuse scattering.*** Thermal diffuse scattering (TDS), or electron-phonon scattering, is important both in STEM and high-resolution TEM (HRTEM) and is responsible for features including diffuse backgrounds and Kikuchi lines as well as for a large part of the annular dark-field signal ^41^. The most common and successful method for simulating TDS with dynamical scattering is the frozen phonon model. Its basic idea relies on a rather classical picture where each electron sees a different configuration of atoms displaced from equilibrium by thermal vibrations. Despite its simplicity, the model’s accuracy has been substantiated both theoretically ^42^ and numerically by comparison to a fully quantum mechanical model in the standard Born–Oppenheimer approximation ^43^.

The frozen phonon structures are usually created by independently displacing each atom according to a Gaussian distribution with a fixed width depending on the element, i.e., following the approximate Einstein model. A more accurate thermal ensemble can be directly generated through molecular dynamics simulations at a given temperature ^44^. In abTEM this is facilitated through the Atomic Simulation Environment (ASE) ^45^, which interfaces with several popular MD codes. abTEM’s example library include a single-worksheet demonstration of using the high-performance classical MD code LAMMPS ^46^ through ASE in conjunction with abTEM.

## 3 Implementation

### 3.1 Python

Most multislice codes have a somewhat rigid character, using a graphical user interface or input files to control the execution of binaries written in compiled languages such as Fortran and C. A distinguishing feature of abTEM is that all tasks are accomplished by writing and running Python scripts. Python is a dynamically typed programming language with clear and expressive syntax. It can be used for writing everything from small scripts to large programs and libraries like abTEM itself. The Python language has continually gained popularity for scientific computing for the past two decades, thanks in particular to its extensive and broad base of open-source libraries. In the TEM community, this includes packages such as H yperS py ^47^ and P y4DSTEM ^48^.

Some multislice codes have a Python or MATLAB scripting interface ^13, 16^, but the level of interactivity rarely goes beyond automation of input and output of fixed simulation modes. Our previous effort in providing a Python interface to the powerful multislice code QSTEM went somewhat further, but the actual simulations were still not performed by P yQSTEM itself ^6^. Now, abTEM relies on external Python libraries only to handle atoms and DFT calculations, or to enhance its numerical performance.

**Figure 1.**
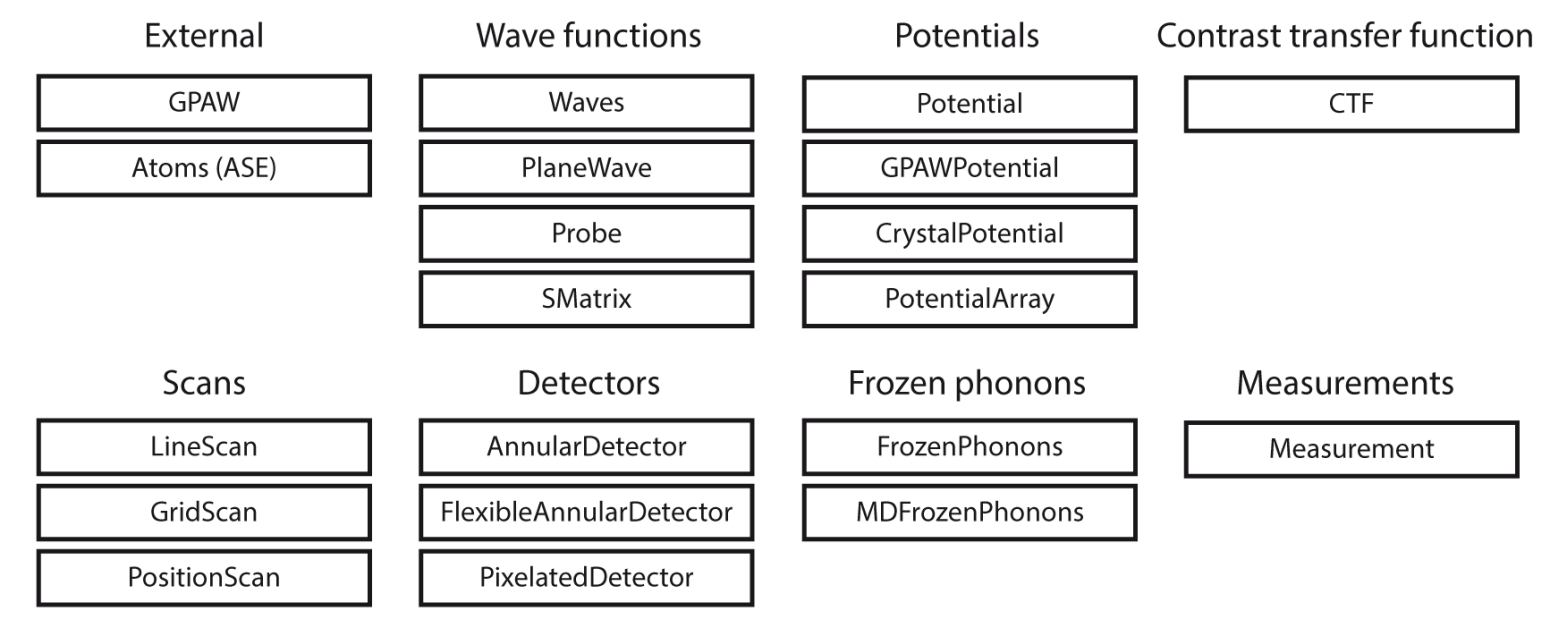
The modular design of abTEM is enabled by Python classes that implement physically meaningful concepts. Consistent object-oriented design allows new instances of these classes to be easily implemented as our code grows or new techniques or instrumentation become available.

abTEM also differs from earlier codes by not directly implementing *any* common imaging modes, but invites the user instead to mix and match objects to construct the desired simulation. The design patterns used by abTEM thus take inspiration from object-oriented scientific codes, particularly ASE ^45^, which is also used for importing and manipulating atomic structures and interfacing with other atomistic simulation codes. The idea behind this approach is that the user operates using understandable concepts from physics instead of computational details. abTEM provides Python classes like Waves that store the wave function as a N umP y array, and implements methods like multislice for propagating the wave function. In Figure 1, we show common objects the user can expect to interact with.

### 3.2 Modularity

A key design principle in object-oriented programming is to keep different objects independent, improving readability and simplifying further development as contributors only need familiarity with a fraction of the codebase. The objects in Figure 1 are divided into categories, and the objects within a category are generally made to be interchangeable. To use similar classes interchangeably in conjunction with others requires that the classes follow a template. A considerable amount of thought has gone into creating templates that allow flexibility to implement new functionality with the minimum effort. Further, since all code is open source in Python, this is highly accessible even for non-expert users.

Each of the classes shown in Figure 1 is a subclass of an abstract base class. Any new class within that category is required to implement some basic methods and properties. For example, any detector should implement a minimum of two methods: the allocate_measurement method for creating an array in memory or as an HDF5 file for storing the results, and the detect method for taking a wave function object and returning a corresponding measurement. A detector following this pattern is automatically compatible with any other algorithm implemented in abTEM.

### 3.3 Performance and dependencies

Dynamic interpreted languages such as Python are attractive for domain experts and scientists trying out new ideas. However, the performance of the interpreter is often a barrier to high performance. To mitigate this, N umP y is one of the most important scientific libraries in the Python ecosystem as it provides a multi-dimensional array ( ndarray) object that has become the foundation of efficient numeric computation in Python. If an operation can be performed with a single call to a N umP y function, the performance matches the underlying implementation in C. However, this is not always possible, and scientific codes often rely on Python C extensions to efficiently implement custom computation. For example, the DFT code GPAW consists of about 90% Python with a small amount of computationally demanding functionality implemented in C.

The process of writing a Python C extension can be error-prone due to the difficulty of manually managing the reference counts of Python objects and generally requires a lot of ‘boilerplate’ code, even for simple use cases. Further, the inclusion of compiled code complicates the release of cross-platform programs, as it requires the user to be familiar with code-compilation or the maintainer to provide precompiled binaries. abTEM achieves high performance without C extensions by using the N umba library ^49^, which is a just-in-time Python compiler that focuses on scientific and array-oriented computing. N umba analyzes and optimizes Python code and then uses the LLVM compiler library to generate machine code with a performance similar to that of C.

Python’s recent popularity in machine learning has led to the release of high-performance libraries for GPU-accelerated calculations. According to our benchmarks, pre-eminent GPU libraries’ speed is largely identical for performance-critical FFT and matrix multiplication operations. Our choice to use C uP y ^50^ comes down to its compatibility with N umpy: C uP y can in most cases be used as a drop-in replacement, while users without access to a compatible GPU can install abTEM without it. Due to C uP y limitations, abTEM currently only supports Nvidia’s CUDA toolkit on Windows and Linux, requiring an NVidia graphics card – all GPU multislice programs currently require CUDA, apart from the STEM cl package ^18^, which uses the non-proprietary OpenCL framework. However, OpenCL is, according to our testing, slower than CUDA. abTEM will soon expand to support AMD ROCm GPUs on Linux following the next release of C uP y, which is expected to deliver AMD support. We hope that support for Apple Silicon will soon follow.

abTEM currently offers only limited multi-CPU/GPU parallelization, requiring the user to distribute the work across multiple CPU/GPU workers and gather the results afterwards. For example, one can assign each worker a fraction of the frozen phonon configurations and add up the results after they are done. Our online repository ^21^ demonstrates multi-CPU parallelization using the commonly available Message Passing Interface (MPI), utilizing the excellent mpi4 py library and parallel HDF5 to support parallelized filesystem access. We expect to further improve parallelization in the near future.

### 3.4 GPU memory management

The arrays used to store the potential or scattering matrix may be too large to fit into limited GPU memory. Hence, recent multislice applications have emphasized the importance of asynchronous memory transfer, whereby memory transfer and kernel execution are performed simultaneously ^16, 18^.

Since we prefer to keep abTEM as simple as possible, we use serial memory transfer and kernel execution. We focus instead on using batching to limit the memory transfers and ensure high GPU utilization. Both FFTW and cuFFT support batch FFTs, whereby multiple Fourier transforms of the same size can be computed simultaneously. Hence, a number of wave functions are propagated simultaneously in the multislice algorithm. For small batch sizes, memory transfer is indeed a considerable overhead, but this generally disappears as the batch size grows. In HRTEM, only a single wave function is propagated, hence batching is not possible; however, in this case there is no memory to transfer, since each slice is only used once and calculated on the fly on the GPU. For large simulations, memory constraints may limit the batch size, but we generally did not find memory transfer to be a significant bottleneck.

## 4 Operation

### 4.1 Minimum system requirements

Although a powerful system is recommended for real work, any modern laptop will meet the minimum requirements for running abTEM. It is also possible to run for teaching and demonstration purposes using free cloud computing services, such as Binder ^51^.

### 4.2 Code examples

To demonstrate the use of abTEM, we show a few basic examples below, with many more found in our online repository online repository ^21^. We start with a simple example showing how to combine objects together for a HRTEM simulation:


from ase.io import read
from abTEM import PlaneWave, CTF
atoms = read('atoms.cif')
plane_wave = PlaneWave(sampling=0.01, energy=300e3)
exit_wave = plane_wave.multislice(atoms)
ctf = CTF(defocus=200, focal_spread=40)
image_wave = ctf.apply(exit_wave)
image = image_wave.intensity()


A structure file is imported as an ASE Atoms object; the read method supports a multitude of popular file formats. The incoming wave function is defined as a plane wave and propagated through the structure using the multislice algorithm, with the default IAM potential implicitly applied. abTEM follows the ASE unit convention with Å used for spatial variables and eV for energies. The wave function is transferred to the image plane using a contrast transfer function (CTF), where the intensity is calculated to obtain the final image. In such functions, the angular unit of mrad is used.

The example could be easily modified to instead simulate electron diffraction by using the diffraction_pattern method as the final step, and further to simulate convergent-beam electron diffraction (CBED) by using Probe to define the incoming wave function. The contrast transfer function includes partial coherence through the widely-used quasi-coherent approximation ^8^; examples of the correct incoherent summation can be found in the online repository.

Next, we show the code for a basic annular dark field STEM simulation:


from ase import Atoms
from abTEM import Probe, Potential, GridScan, AnnularDetector
atoms = Atoms('C')
atoms.center(vacuum=10)
potential = Potential(atoms, slice_thickness=0.5)
probe = Probe(sampling=0.01, energy=300e3, semiangle_cutoff=30)
scan = GridScan(start=(0,0), end=potential.extent, sampling=0.1)
detector = AnnularDetector(inner=70, outer=180)
adf_signal = probe.scan(scan, detector, potential)


This example creates a single C atom at the center of a 10 *×*10 *×*10 Å simulation cell. Whereas previously the default method for calculating the potential was used, we explicitly set the Potential object to define how this is done. The incoming wave function is now defined as a probe, and for detecting the exit wave functions, an annular dark field detector is defined. Using a PixelatedDetector in place of an AnnularDetector can change a simulation from annular dark field STEM to 4D-STEM, and the PRISM algorithm could be used instead of multislice by simply replacing the Probe object with SMatrix.

The downside of our approach is that it may not be immediately obvious for new users how to combine the objects to achieve the desired simulation mode. To assist these users, the abTEM repository contains an expanding example library implementing common (and some less common) image simulations. Users can download the notebooks and modify them; this could be as easy as changing the imported structure file.

### 4.3 Cross-platform interactive notebooks

Given the visual nature of image simulations, a particularly attractive way of using abTEM is through Jupyter notebooks. This web application allows users to create and share documents that contain live code, visualizations and narrative text. The notebooks are composed of cells, each of which may contain the code for accomplishing a subtask. To facilitate a visual workflow, most of the classes in abTEM implement a method for creating a quick visualization of each subtask; for example a heatmap of the projected potential or the profile of an electron probe. The mix of code, explanatory text and visualizations immediately provides documentation of the simulation, allowing others to understand and reproduce it.

Thus, abTEM is designed to be used via scripts, in an interactive Python session, or in a Jupyter notebook. Since these are cross-platform tools, support for popular operating systems including Linux, MacOS and Windows is easy to offer and maintain. Nonetheless, we recognize that this is not always the optimal way of interacting with code. Relying on new tools from the data science community developed for interactive data analysis, abTEM also supports the creation of small web applications embedded in a Jupyter notebook.

For example, the apply method of the CTF class has an “interact” keyword that, if set to true, opens an interactive visualization of the resulting image. The ability to change the defocus and spherical aberrations using a simple slider and seeing the image update provides quick intuitive understanding of the effects and interplay of the parameters.

The tools for creating notebook-embedded interactive visualizations can with little effort also be used for creating simple web applications that are straight-forward to deploy on a server. This could be useful for teaching, as it enables users to experiment with simulating TEM images with only a web browser.

## 5 Use cases

Finally, we turn to realistic simulation use cases. These calculations were performed using a consumer-grade desktop PC running Linux (Ubuntu 20.04.1) equipped with an 8-core Intel Core i9-9900K processor and 32 GB of memory and a NVidia RTX 2080 Ti graphics card with 2944 cores and 11 GB of graphics memory. Jupyter notebooks for completely recreating each example are available online, where we plan to collect all publication-related open code.

### 5.1 Comparing IAM to *ab initio* for hBN

In our first example, we illustrate the effect of valence bonding by comparing IAM and DFT potentials for the simulation of hexagonal boron nitride (hBN), whose strong ionicity makes it an ideal test case. As has been previously noted, while annular dark field (ADF) contrast is rather insensitive to bonding, both bright field (BF) contrast (previously measured using HRTEM ^2^) and electron diffraction (ED) intensities (measured with parallel illumination HRTEM ^6^) do show marked quantitative differences; very recently, this was also confirmed by ptychography ^52^. abTEM makes such comparisons extremely easy to make, not to mention facilitating the use of *ab initio* potentials in the first place.

We create an orthogonal periodic unit cell of hBN using the ASE constructor for graphene with the lattice constant of hBN and replacing the C atoms with B and N, and create both IAM and DFT scattering potentials, the latter requiring a cheap GPAW plane wave calculation for converging the valence electron density. We then simulate a STEM experiment at an electron energy of 80 keV and a probe convergence semi-angle of 31.5 mrad, with two detectors: a BF detector with a semi-angular range of 0 to 16 mrad, and a high-angle ADF detector for the range 95 to 126 mrad.

We scan over the entire cell at the Nyquist sampling for the highest computational efficiency, here corresponding to a real-space spacing of 0.3 Å, allowing us to interpolate the measured images down to the sampling of the potential (0.04 Å) without introducing artifacts. Images can further be easily tiled to display larger fields of view or calculate diffraction patterns, and interpolated line profiles plotted to facilitate quantitative comparisons.

**Figure 2.**
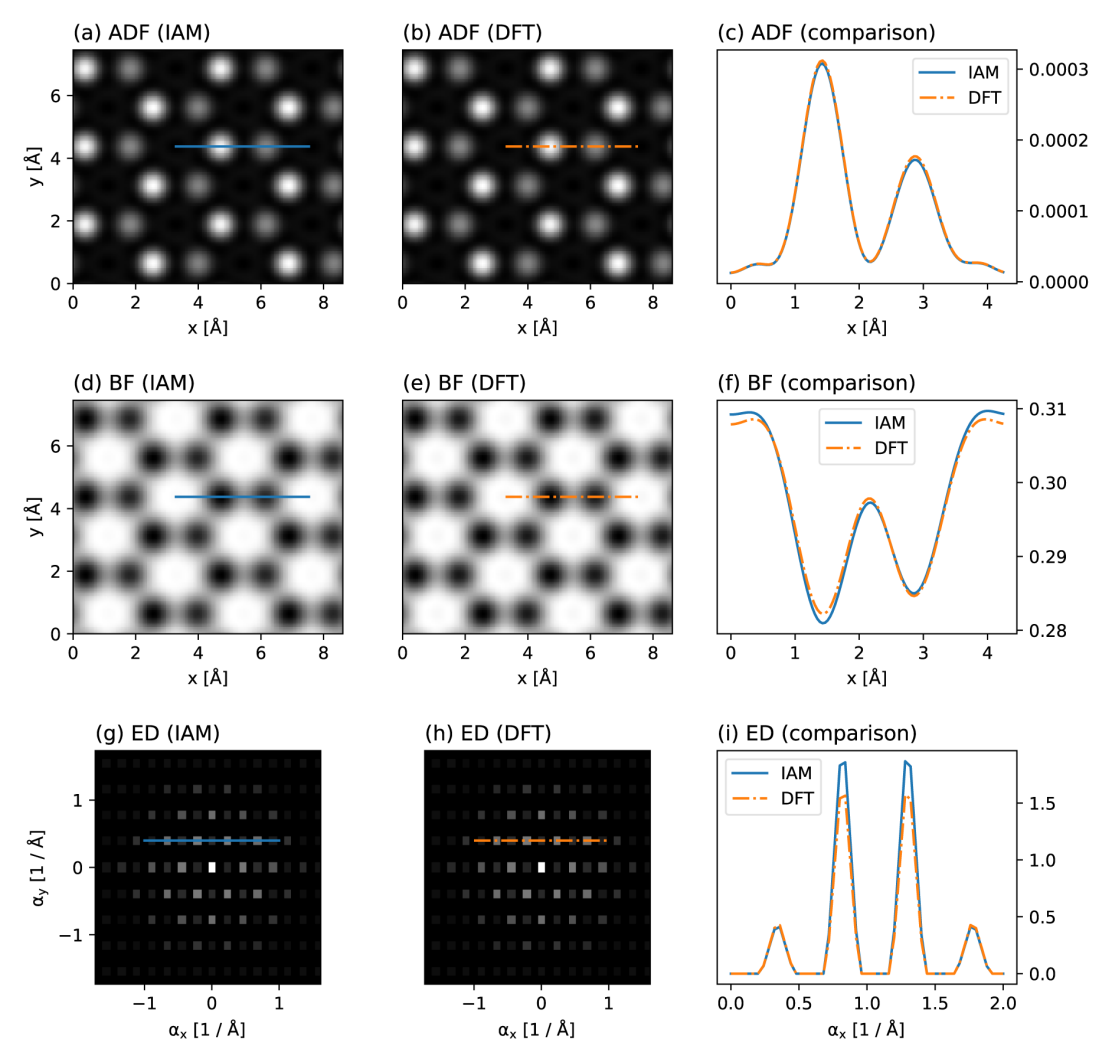
Comparison of independent atom model (IAM) and density functional theory (DFT) scattering potentials for hBN. Annular dark-field (ADF) images based on the ( **a**) IAM and ( **b**) DFT, with ( **c**) line profiles plotted over the N and B sites. ( **d**, **e**) Corresponding bright-field (BF) images and ( **f**) line profiles. ( **g**, **h**) Corresponding electron diffraction (ED) patterns displayed on a logarithmic scale derived from tiled images, and ( **i**) line profiles through the first two diffraction orders. While the ADF contrast is nearly completely insensitive to valence bonding, both BF contrast (see Ref. 2 for an experimental measurement) and the ED intensities (see Ref. 6 for an experimental measurement) do show a marked difference due to charge redistribution in this highly ionic compound that is not described by the IAM.

Figure 2 shows our simulated results. As expected, the ADF contrast is almost entirely insensitive to the potential, but a reduction of the N atom scattering intensity by about 5% due to charge redistribution is visible in the BF contrast. The same effect also reduces the intensity of the first diffraction order in the simulation based on the DFT potential. Thus, as is being increasingly recognized ^22^, effects due to charge redistribution can be measured using modern TEM methods – and readily simulated using abTEM.

### 5.2 4D-STEM ptychography of MoS _2_

In our second example, we attempt to computationally reproduce recent 4D-STEM experiments on MoS _2_, where different measurements are reconstructed from a fast pixelated direct electron detector ^53^. The modular design of abTEM makes it easy to directly model 4D-STEM experiments, and we have implemented both integrated center of mass (iCoM) measurements ^54^ and the popular ePIE phase reconstruction algorithm ^55^ in Python to facilitate such simulations.

We create a periodic orthogonal 4 *×*4 *×*1 MoS _2_ supercell, and create a vacancy by deleting one S atom from the top layer. We then create an IAM scattering potential and a scanning probe with parameters corresponding to the experiment ^53^: an electron energy of 80 keV, probe convergence semi-angle of 21.4 mrad, defocus corresponding to the given chromatic aberration parameters, a reasonable amount of residual spherical aberration and focal spread, and some residual astigmatism to try and mimic the experimental contrast. The scan area is chosen to closely reproduce the experimental field of view.

We define two PixelatedDetectors with different spatial samplings (0.21 Å for BF, ADF, and iCoM, and 0.45 Å for ptychography). The Experimental signal is emulated using Poisson noise with an electron dose of 6 *×*10 ^6^ e ^–^ / Å2. Additionally, we define a BF detector with a semi-angular range of 0 to 21.4 mrad and an ADF detector for the range 64.2 to 85.6 mrad, which are post-integrated from the 4D-STEM measurement alongside the iCoM signal. For the phase reconstruction, we run five iterations of the ePIE algorithm. Diffractograms are calculated from the measured images using a built-in method.

**Figure 3.**
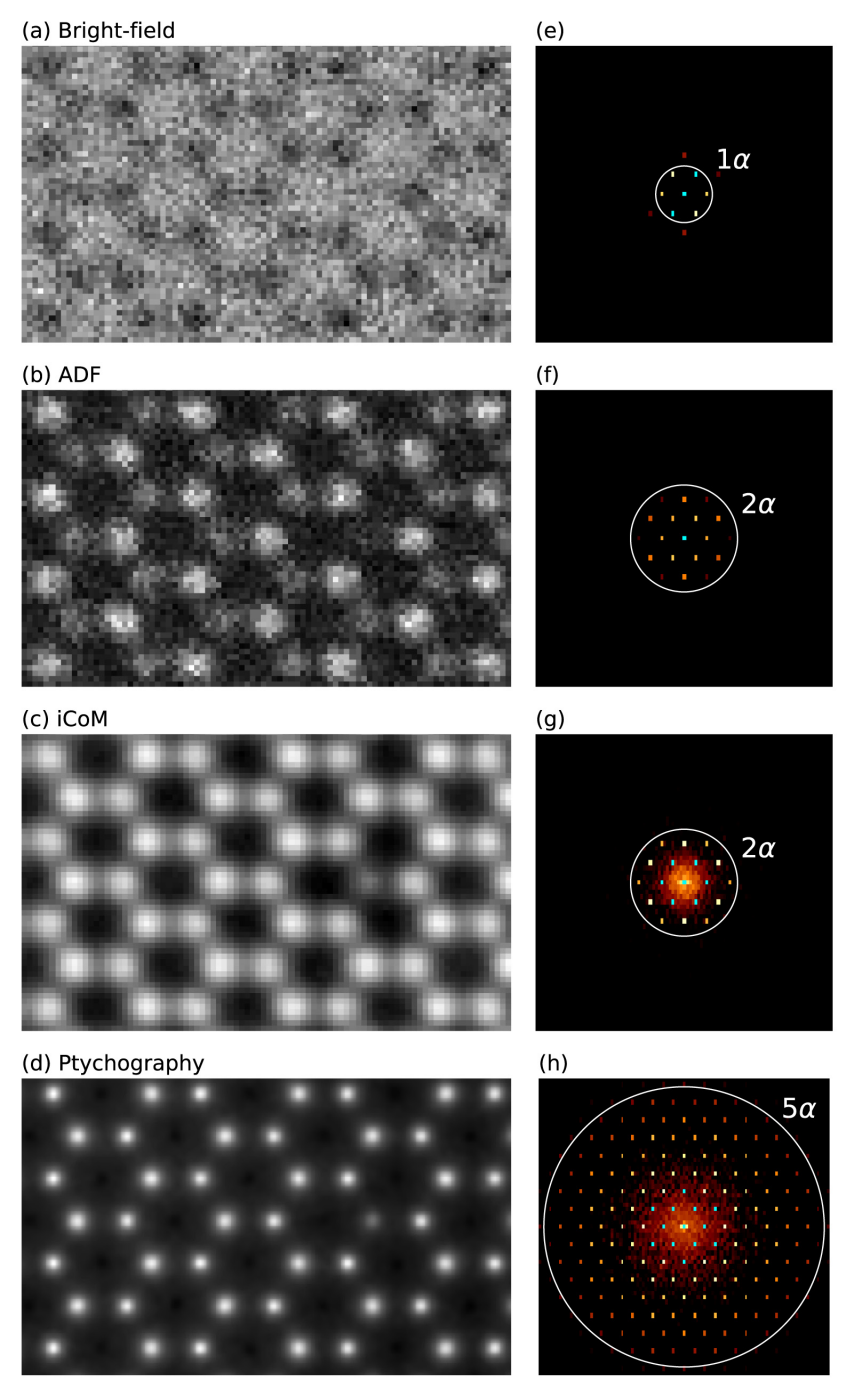
4D-STEM simulations of MoS _2_ with a single S vacancy. ( **a**)–( **c**) Bright-field, annular dark field (ADF), and integrated center of mass (iCoM) (0.21 Å sampling) measurements and ( **d**) ptychographic phase reconstruction (0.45 Å sampling), all derived from pixelated detector measurements with applied Poisson noise. ( **e**)–( **h**) Diffractograms displayed on logarithmic scale calculated from the images and colored with the color map ‘afmhot’ with a cyan color set for the brightest values. For the experimental work that inspired this example, see Ref. 53.

Figure 3 shows our simulated results, which mimic the experimental Figure 2 in Ref. 53. According to our testing, for a rigid semiconducting material such as MoS _2_, neither the inclusion of structural relaxation or using an *ab initio* electrostatic potential would affect the results significantly. Note that we did not apply any thermal averaging in this simple example, which would reduce the contrast of S _2_ columns compared to the S vacancy, bringing the results into better agreement with the experimental data ^53^.

### 5.3 CBED and ED with MD phonons

In our third example, we examine the effect of phonon correlations on TDS in CBED and ED patterns. The example demonstrates how abTEM can be used in conjunction with a MD code to calculate accurate thermal ensembles for use in a frozen phonon calculation. The example also demonstrates the performance of abTEM with respect to imaging modes that only require a single wave propagation, and hence where calculating the IAM potential is a substantial part of the overall computational cost.

We create a sample structure of crystalline silicon in the [100] zone axis, with a 200 *×*200 *×*1303 Å ^3^ supercell containing 2.6 million atoms. The thermal ensemble is simulated using molecular dynamics with the Tersoff ^56^ force field implemented in LAMMPS, and periodic boundary conditions are applied in all directions. We obtain 16 phonon snapshots after equilibration at 300K for 50 ps, chosen at time intervals exceeding the expected phonon correlation time (10 ps). The available four rotation and two inversion symmetries are used to effectively simulate a total of 128 configurations.

The wave function is sampled at 0.025 Å/pixel and the potential is sliced with a thickness 0.1 Å, corresponding to sampling the potential on a voxel grid of size 4096 *×*4096 *×*13000. The slices are chosen so thin to accurately portray features that depend on the 3D symmetry of the crystal. This could also be accomplished by slicing the potential at the crystal planes ^57^, but that would neglect the 3D nature of the phonons. The potential is calculated on-the-fly using standard infinite projection integrals. On GPU, a batch size of six slices is automatically selected by the program, whereas all CPU threads are already fully engaged without slice parallelization.

The multislice simulation of each frozen phonon configuration took 182 s on GPU and 85 min on CPU, not including the MD simulation. The time spent on calculating the potential and wave propagation was approximately equal, as was reported in the description of the potential algorithm ^11^. Note that in this comparison, the calculation of the transmission function from the potential is counted as wave propagation.

**Figure 4.**
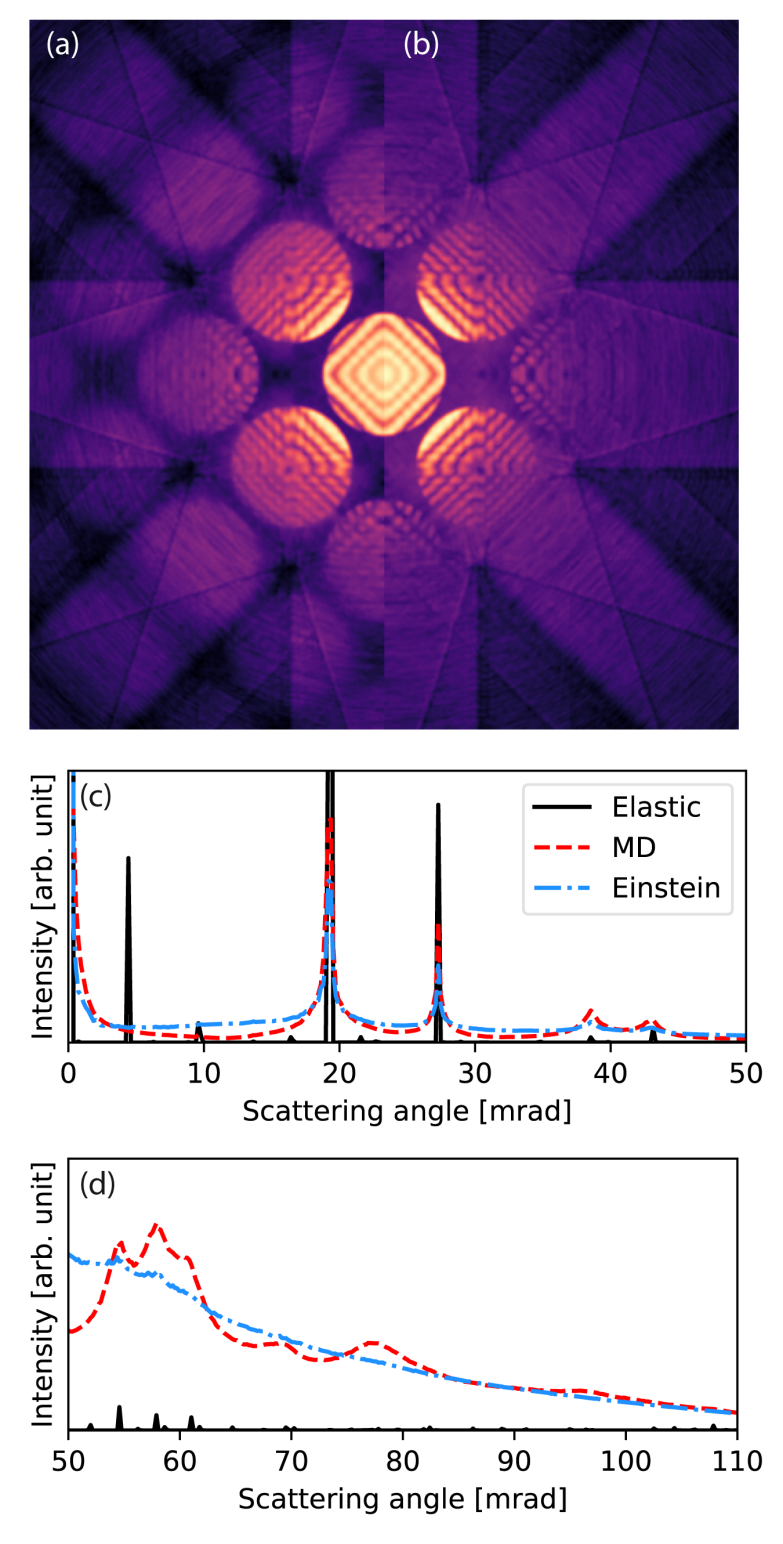
Convergent-beam electron diffraction (CBED) simulation with thermal diffuse scattering. ( **a**)–( **b**) Calculated CBED patterns for Si[100] at 100 keV with an aperture of 9.4 mrad. ( **a**) Calculated using molecular dynamics (MD) and ( **b**) calculated using an Einstein model. In ( **a**) more of the higher order disks are visible. ( **c**)–( **d**) The rotationally averaged electron diffraction patterns for Si[100] at 100 keV.

For comparison, we calculate the equivalent CBED pattern using the Einstein model. The standard deviation of the Gaussian distributions of each atomic position are set to 0.126 Å to match that found for the MD thermal ensembles. Figure 4 shows that the Einstein model and the more accurate MD model are quantitatively different, as has been discussed before ^44, 58^. The correlation of atomic vibrations in the crystal results in more scattering around the Bragg reflections, and the Einstein model overestimates the low-angle diffuse background. At medium-to-high scattering angles the Einstein model provides good agreement with the MD result, whereas a fully elastic model without any phonon images severely underestimates high-angle scattering.

### 5.4 Large-scale STEM simulations

In our final example, we show how abTEM is capable of performing extremely large STEM simulations using the PRISM algorithm. We examine the speed of this algorithm compared to multislice, as well as the speed of abTEM’s pure Python PRISM algorithm to P rismatic, which is an open-source C++/CUDA package using asynchronous memory transfer. To our knowledge, of the two existing codes implementing PRISM, P rismatic and P yM ultislice ^16, 19^, the former released by the original developers of the algorithm is faster.

We simulate a STEM image of a decahedral gold nanoparticle with 20324 atoms. The supercell is 136 *×*136 *×*118 Å ^3^, and a grid of 4096 *×*4096 pixels was used, corresponding to a real-space sampling of 0.033 Å or maximum anti-aliased scattering angle of 419 mrad; the slice thickness was 1.0 Å. The convergence semi-angle is set to 25 mrad, thus this probe requires a basis set of 20849 plane waves to represent (or more than 1 Tb of memory) and hence interpolation is required. We set the interpolation factor to 16, resulting in a basis set of just 81 plane waves, with an effective probe window of 8.5 *×*8.5 Å ^2^ and a maximum error of 1.2% compared to standard multislice, which is about the same error as is introduced by using 1.0 Å as opposed to 0.5 Å slices.

The probe is scanned at the Nyquist frequency (here 0.42 Å per pixel), resulting in a total of 106276 probe positions. The calculations took 73 s per frozen phonon configuration on GPU and 1224 s on CPU. The scattering matrix was stored in CPU memory in both calculations and the potential was calculated on the fly, as keeping both the potential and scattering matrix in memory was not possible. For comparison, using multislice the same calculation took around 96000 s ( *∼*1 day) on GPU and 2220000 s ( *∼*25 days) on CPU, though with a precalculated potential. This shows that PRISM was 1315 times faster on GPU and 1813 times on CPU, in line with the findings of the original authors ^20^.

The PRISM algorithm requires two steps: propagating the scattering matrix using the multislice algorithm and reducing the scattering matrix at each probe position. In all of the calculations above, the multislice propagation was the more expensive step. For large simulations, this step is unlikely to suffer much from the overhead of calling the FFTW and cuFFT libraries from Python and hence not suffer from our pure Python approach. It is also difficult to see how to significantly further improve the performance of this step.

**Figure 5.**
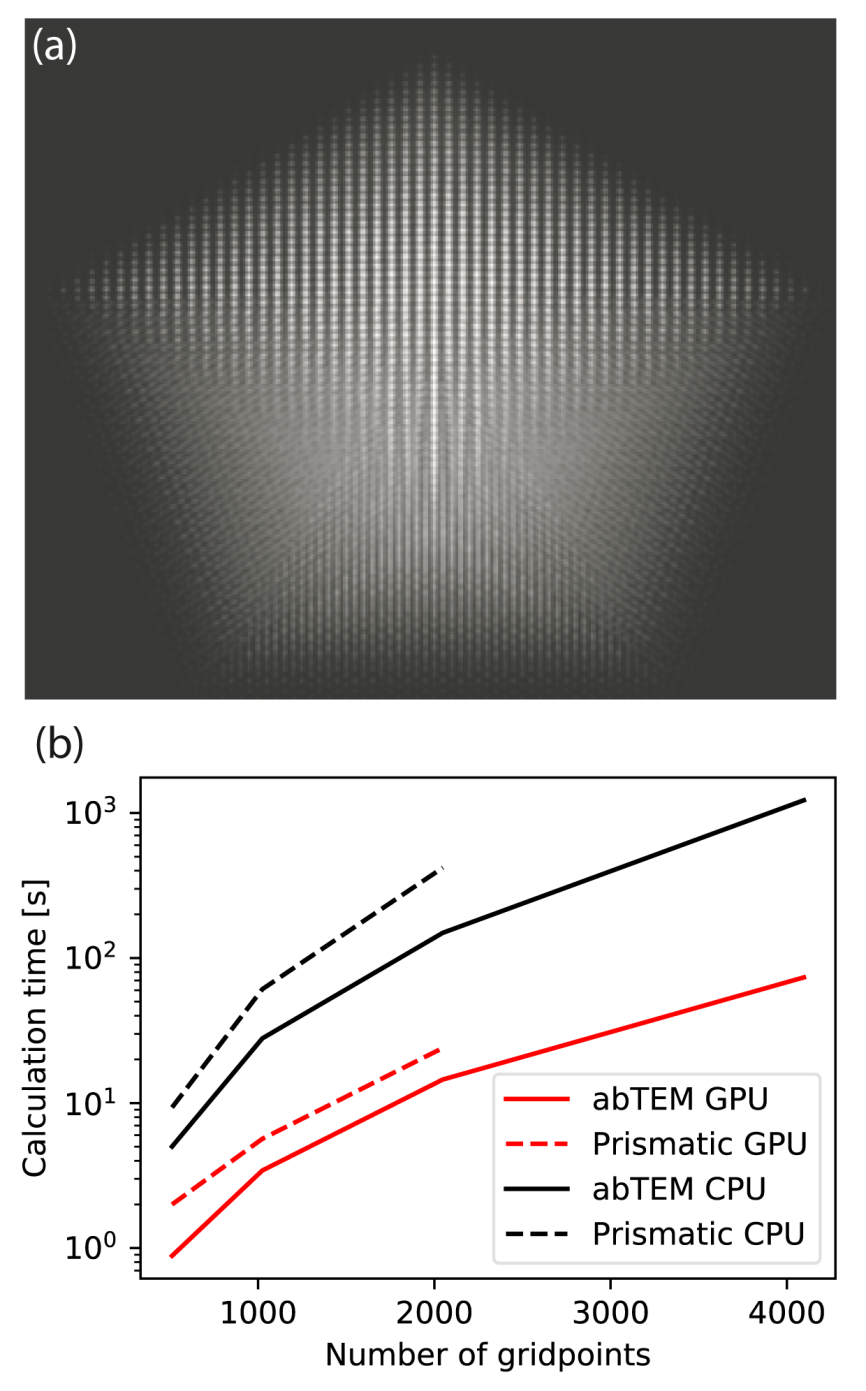
( **a**) High-angle annular dark-field STEM image of a decahedral nanoparticle consisting of more than 10 ^5^ atoms, simulated in 15 s using abTEM’s GPU-accelerated PRISM algorithm. ( **b**– **c**) The time required for simulating a nanoparticle as a function of the size of system. ( **b**) Calculation time for simulating a decahedral nanoparticle, as a function of system size given as as the number of pixels on each side of the simulation grid. The calculation for the largest nanoparticle is left out for Prismatic as this simulation was not possible due to running out of memory.

In Figure 5, we benchmark abTEM against P rismatic for the simulation of differently sized decahedral nanoparticles. The size of the simulation supercell was varied, while keeping the real space sampling and number of plane waves in the probe constant at the values given above. abTEM was consistently up to twice as fast compared to P rismatic on our benchmark system using either GPU and CPU algorithms.

Note that P rismatic uses a factor of 2 antialiasing aperture, while abTEM uses a factor of 3 */*2. Hence, the maximum scattering angle in abTEM is 25% higher. It also has the ability to calculate the potential on the fly, whereas P rismatic always precalculates the potential: the simulation of largest nanoparticle system shown in Figure 5(a) was not possible using P rismatic on our hardware as the potential could not fit into memory. We do acknowledge that P rismatic has multi-CPU and -GPU support, and thus is likely to outperform abTEM on other hardware. Nonetheless, it is clear from this comparison that abTEM performs exceedingly well despite its pure Python implementation.

## 6 Conclusion

We have presented a new multislice simulation code called abTEM. The program was created to integrate atomistic simulation codes with the multislice algorithm in order to meet the demands of improved accuracy and flexibility posed by ongoing experimental advances. abTEM is written entirely in Python and has all the benefits that entail, and we have demonstrated that it is as fast as a highly optimized C++ code thanks to effective use of modern open-source software libraries. Python is also becoming important on the experimental side thanks to the Nion S wift microscope control software, enabling full integration of all aspects of the modern TEM research workflow. Notably, Python scripting has also been recently enabled for the Gatan Microscopy Suite ^59^.

abTEM is under active open development and is made to be easily extendable. We expect to significantly improve multi-CPU and -GPU parallelization in the near future, including support for AMD graphics cards following the next release of C uP y. In terms of new simulation modes, our current focus is on improving the description of inelastic energy loss, and we are specifically working on implementing core losses following the work of Brown ^40^ and phonon losses following that of Zeiger and Rusz ^37^. We have also taken some initial steps towards implementing plasmon losses following the approach of Mendis ^39^.

The code is now fully functional and ready for scientific work. We hope to entice experts in the field to try it out and join us in implementing new features, but are especially keen to enable experimentalists to complement their work by easily accessible simulations. Our ultimate goal is a fully *ab initio* description of all aspects of TEM simulation.

## Data availability

All data underlying the results are available as part of the article and no additional source data are required.

All presented figures can be reproduced with the open code available from:

https://github.com/jacobjma/abTEM/tree/master/articles/2021_ORE_abTEM ^21^.

## Software availability

Documentation and code examples available from: https://abtem.readthedocs.io/en/latest/index.html

Source code available from: https://github.com/jacobjma/abTEM

Archived source code at time of publication: https://doi.org/10.5281/zenodo.4767986 ^21^.

License: GNU General Public License version 3
