# Supplementary material for: The abTEM code: transmission electron microscopy from first principles
Source: Open Res Eur. 2021 May 21;1:24. Originally published 2021 Mar 24. [Version 2] doi: 10.12688/openreseurope.13015.2 (PMC10446032; doi:10.12688/openreseurope.13015.2)
Supplement: Peer review history

# Peer review: Reviewer response for version 2

**DOI:** 10.21956/openreseurope.14825.r26947

**Published:** 2021-05-21

**Authors:** Colin Ophus

**Affiliations:** 1 National Center for Electron Microscopy, Molecular Foundry, Lawrence Berkeley National Laboratory, Berkeley, CA, USA

All of my comments have been addressed. Great paper!

Is the rationale for developing the new software tool clearly explained?

Yes

Is the description of the software tool technically sound?

Yes

Are sufficient details of the code, methods and analysis (if applicable) provided to allow replication of the software development and its use by others?

Yes

Is sufficient information provided to allow interpretation of the expected output datasets and any results generated using the tool?

Yes

Reviewer Expertise:

Transmission electron microscopy, electron scattering simulation, data analysis, materials science.

I confirm that I have read this submission and believe that I have an appropriate level of expertise to confirm that it is of an acceptable scientific standard.

# Peer review: Reviewer response for version 1

**DOI:** 10.21956/openreseurope.14085.r26646

**Published:** 2021-04-09

**Authors:** Hamish Brown

**Affiliations:** 1 Advanced Microscopy Facility, Bio21 Molecular Science and Biotechnology Institute, University of Melbourne, Melbourne, Australia

The field of TEM image simulation is now very mature with many free and open source simulation codes available. However many of these codes are written in low-level languages such as Fortran and C++ which means that they can be inflexible and difficult to modify, especially for the average microscopist who is not an expert programmer. Additionally there has been insufficient research on developing workflows for including the electrostatic potential of materials generated by "first principals" methods such as density functional theory (DFT) and molecular dynamics in TEM simulations. This paper addresses both of these challenges by introducing a Python TEM simulation toolkit that is straight-forward to use, implements more recent advances such as the reciprocal-space interpolated scattering matrix (PRISM) approach and Tanh-Sinh quadrature for the correct treatment of 3D potentials and interfaces very conveniently with the GPAW DFT library and the LAMMPS molecular dynamics packages.

 The paper well written, with clear and concise discussions of the code furnished with instructive and relevant examples. The code itself is a well finished product and is intuitive in the way that it is put together with Python notebooks providing useful examples of how to run key simulations. I whole heartedly recommend indexing of the work with only a few minor points I would like to see addressed:

 In the introduction suggest replacing "reaching nowadays sub-Ångström resolution" with "with sub-Ångström resolution now routinely achievable in modern aberration corrected instruments"

 In the second to last paragraph of sec. 1, suggest replacing "such succession" with "Fourier transform"

 In sec. 2.4 the brief description of the PRISM algorithm is a little vague (understandable given the complexity of the concept). Suggest changing to "the electron probe can be expressed using a plane-wave basis and the individual plane waves are propagated through the specimen using a pre-calculated scattering matrix and coherently summed at the exit surface to calculate the scattered probe". In the next sentence I suggest modifying "are then multiplied by a coefficient $\alpha_{nm}$" by being explicit that $\alpha_{nm}$ is a complex component of the scattering matrix. A short sentence explaining why the introduction of a PRISM interpolation factor leads to a tiling out in real space, where keeping only every fth coefficient in Fourier space is equivalent to creating an f x f tiling of that function (I believe this is referred to as periodic summation) and hence the need for real space cropping, would be useful.

 In sec 4.2 Defocus spread of conventional TEM images can be taken into account by integrating the measured intensity for a given defocus over the probability distribution of the defocus spread. See for example Eq. (3.20) in Kirkland's textbook, reference 8 in the manuscript. However the first code example of 4.2 seems to apply this spread to the complex electron wave function before the intensity is evaluated. For a weak phase object the two approaches should be equivalent however this is not true in general. Is this another example the quasi-coherent approximation discussed with respect to partial coherence earlier in this chapter?

 In Sec 5.1 I was initially alarmed by the sentence "We create an orthogonal periodic unit cell of hBN using the ASE constructor for graphene", inspection of the code (hBN_DFT_IAM.ipynb) convinced me that indeed the graphene constructor is only used to create a unit cell structure, since graphene shares the symmetry of hBN, and the atoms and unit cell are modified post-hoc to reflect the correct atomic structure of hBN. Suggest mentioning this in the text.

 In section 5.2, I am curious as to how well probe correction worked in this simulated example. If the aberrations in the probe are discernable by eye it would be instructive to place the aberrated probes used in the simulation and reconstructed next to the ptychographic reconstruction.

 In sec 2.3.2 the authors discuss how Tanh-Sinh quadrature is used to accurately evaluate integrals over the z coordinate, this was first discussed in the context of TEM simulation by Lobato and Van Dyck (reference 13 in the current manuscript) so work should also be referenced in this section.

 In reference 48. the author name bsavitzky seems to be inappropriately formatted

Is the rationale for developing the new software tool clearly explained?

Yes

Is the description of the software tool technically sound?

Yes

Are sufficient details of the code, methods and analysis (if applicable) provided to allow replication of the software development and its use by others?

Yes

Is sufficient information provided to allow interpretation of the expected output datasets and any results generated using the tool?

Yes

Reviewer Expertise:

TEM simulation and experiment, condensed matter physics.

I confirm that I have read this submission and believe that I have an appropriate level of expertise to confirm that it is of an acceptable scientific standard.

# Response

**Published:** 2021-05-18

**Authors:** Toma Susi

Original comments by the reviewer are reproduced in *italics *below, followed by our point-by-point replies.

*The field of TEM image simulation is now very mature with many free and open source simulation codes available. However many of these codes are written in low-level languages such as Fortran and C++ which means that they can be inflexible and difficult to modify, especially for the average microscopist who is not an expert programmer. Additionally there has been insufficient research on developing workflows for including the electrostatic potential of materials generated by "first principals" methods such as density functional theory (DFT) and molecular dynamics in TEM simulations. This paper addresses both of these challenges by introducing a Python TEM simulation toolkit that is straight-forward to use, implements more recent advances such as the reciprocal-space interpolated scattering matrix (PRISM) approach and Tanh-Sinh quadrature for the correct treatment of 3D potentials and interfaces very conveniently with the GPAW DFT library and the LAMMPS molecular dynamics packages.*

*The paper well written, with clear and concise discussions of the code furnished with instructive and relevant examples. The code itself is a well finished product and is intuitive in the way that it is put together with Python notebooks providing useful examples of how to run key simulations. I whole heartedly recommend indexing of the work with only a few minor points I would like to see addressed:*

 We thank you very much for your evaluation and constructive comments. We provide point-by-point responses below.

*In the introduction suggest replacing "reaching nowadays sub-Ångström resolution" with "with sub-Ångström resolution now routinely achievable in modern aberration corrected instruments"*

*In the second to last paragraph of sec. 1, suggest replacing "such succession" with "Fourier transform"*

 Thank you for these suggestions, the text has been revised.

*In sec. 2.4 the brief description of the PRISM algorithm is a little vague (understandable given the complexity of the concept). Suggest changing to "the electron probe can be expressed using a plane-wave basis and the individual plane waves are propagated through the specimen using a pre-calculated scattering matrix and coherently summed at the exit surface to calculate the scattered probe". In the next sentence I suggest modifying "are then multiplied by a coefficient $\alpha_{nm}$" by being explicit that $\alpha_{nm}$ is a complex component of the scattering matrix. A short sentence explaining why the introduction of a PRISM interpolation factor leads to a tiling out in real space, where keeping only every fth coefficient in Fourier space is equivalent to creating an f x f tiling of that function (I believe this is referred to as periodic summation) and hence the need for real space cropping, would be useful*.

 We agree that the section was difficult to understand for readers without prior knowledge of PRISM. We have now expanded it, incorporating many of the suggestions. We would argue that the scattering matrix, as defined in the PRISM paper, is just the set of exit waves produced by running the multislice algorithm on the set of plane waves present in the probe-forming aperture, and hence the coefficients, $\alpha_{nm}$, are not as such components of the scattering matrix; this definition is slightly different from the original concept of the scattering matrix. But this is perhaps more a matter of semantics.

*In sec 4.2 Defocus spread of conventional TEM images can be taken into account by integrating the measured intensity for a given defocus over the probability distribution of the defocus spread. See for example Eq. (3.20) in Kirkland's textbook, reference 8 in the manuscript. However the first code example of 4.2 seems to apply this spread to the complex electron wave function before the intensity is evaluated. For a weak phase object the two approaches should be equivalent however this is not true in general. Is this another example the quasi-coherent approximation discussed with respect to partial coherence earlier in this chapter?*

 Yes, the quasi-coherent approximation is applying the focal spread to the wave function, which only works for a relatively small focal spread; abTEM is essentially using Eq. 3.37 in Kirkland’s textbook. We have uploaded an example exploring the accuracy of the quasi-coherent approximation, where we see that it may be adequate for HRTEM, but, of course, very inadequate for CBED. It is possible, although currently a little complicated, to simulate partial coherence using the correct incoherent summation. https://github.com/jacobjma/abTEM/blob/master/examples/other/quasicoherent_approximation.ipynb https://github.com/jacobjma/abTEM/blob/master/examples/other/partial_coherence_4DSTEM.ipynb

*In Sec 5.1 I was initially alarmed by the sentence "We create an orthogonal periodic unit cell of hBN using the ASE constructor for graphene", inspection of the code (hBN_DFT_IAM.ipynb) convinced me that indeed the graphene constructor is only used to create a unit cell structure, since graphene shares the symmetry of hBN, and the atoms and unit cell are modified post-hoc to reflect the correct atomic structure of hBN. Suggest mentioning this in the text.*

 This was admittedly confusing, but luckily as stated, the code is clear on this point. We have now also updated the text accordingly.

*In section 5.2, I am curious as to how well probe correction worked in this simulated example. If the aberrations in the probe are discernable by eye it would be instructive to place the aberrated probes used in the simulation and reconstructed next to the ptychographic reconstruction*.

 In this simple example, our focus was on showing how easy it is to reproduce an experimental dataset from the literature with multiple detectors, and to create a corresponding purely simulated image. Thus we prefer not to add further information into that figure. However, we did use a probe_guess for ePIE that discarded the (in the simulation) known aberrations, but it did not appreciably change the final outcome whether we supplied full knowledge of the probe to the reconstruction or not.

*In sec 2.3.2 the authors discuss how Tanh-Sinh quadrature is used to accurately evaluate integrals over the z coordinate, this was first discussed in the context of TEM simulation by Lobato and Van Dyck (reference 13 in the current manuscript) so work should also be referenced in this section*.

 Thank you for pointing out this significant oversight, the work by Lobato and Van Dyck has now been properly referenced.

*In reference 48. the author name bsavitzky seems to be inappropriately formatted*

 Good catch, it has been fixed.

# Peer review: Reviewer response for version 1

**DOI:** 10.21956/openreseurope.14085.r26649

**Published:** 2021-04-09

**Authors:** Colin Ophus

**Affiliations:** 1 National Center for Electron Microscopy, Molecular Foundry, Lawrence Berkeley National Laboratory, Berkeley, CA, USA

In this manuscript, Madsen and Susi present their image simulation code abTEM for transmission electron microscopy. This code has many things to recommend it:

- completely open source
- all python implementation for easy understanding and modification
- GPU acceleration for fast calculations
- support for calculating the electrostatic atomic potentials from first principles (to include bonding, charge transfer, etc.) through the GPAW DFT code.
- support for atomic cell construction and manipulation via the ASE code.
- implementation of both classic algorithms such as multislice and newer methods like PRISM.

This paper is very well written, and the codes are well documented and user-friendly. I had a lot of trouble in windows, but the binder notebooks all appear to work well. I tested as many of the paper use cases as I could – all worked well, though it would be helpful for the authors to include more sample code for different sample geometries. I should note that I attempted to break the code by checking sampling densities and slice thicknesses that were far outside the range of ordinary simulation parameters (both too large and too small), and I was unable produce results that seemed unphysical – this shows the robustness of the potential integration pipeline developed in this code.

 I also think the implementation of PRISM on the GPU is very clever – the batching of adjacent probes is a very clean way to efficiently use however much GPU RAM is available. It’s also pretty cool to see an all-python GPU implementation that is faster than Prismatic! 

 In summary, I recommend indexing of this work after minor revision. Below I have listed a few questions I have about the paper as well as minor comments that the authors are free to ignore.

 Questions

- “ … allowing us to interpolate the measured images down to the sampling of the potential (0.04 Å) …” How is the interpolation performed? Fourier space padding I assume? If it’s real space, even bicubic interpolation may not be accurate enough.
- The images in Figure 2 look very strange to me. I checked the aspect ratio and it is correct (though it’s quite strange to label one axis in steps of 2.0 and the other in steps of 2.5 Angstroms). However, the atoms are definitely not round, which I assume is due an interpolation error. This can be seen if I saturate the image and draw a perfect circle:
Link to image
- I also don’t quite understand the results in Figure 2 d, e, f. The largest deviation in the BF signal is in the middle of the BN rings, furthest from the atomic potentials – I can rationalize this since this probe position is strongly affected by the degree of ionic screening. However, I would still like to see the quantitative intensity measurements, i.e. dimensions on the vertical axes of b, e, h in units the probe intensity.
- Figure 5a caption or the paper text should probably mention the image is HAADF (I assume) with the detector annular ranges.

Minor suggestions

- Figure 1 shows most of the important objects in abTEM. I would suggest adding a few more: the center-of-mass (along x and y direction) of the probe output intensity would make it easy to perform differential phase contrast (DPC) simulations (which might be already included as an output since DPC results are shown later in the paper). I would also like to see some kind of method for outputting a thickness series – it would be useful for researchers who wish to compare HRTEM images, single STEM probes, or PACBED images with experiments. Finally, it might be useful to include some kind of post-processing as a class, for example numerical integration over a range of angles or defocus values to model limited coherence (maybe this would belong in the Measurement class?).
- Is the choice to go with MPI fixed? I might recommend Dask instead of (or in addition to) since it seems to be easier to scale from local parallelization to large scale HPC parallelization.
- “… is encoded in the scattering of the electron waves by the electrostatic potential of the specimen.” The electron wave can also scatter from electrostatic and magnetic fields too. I am particularly interested in using abTEM to simulate samples with significant charge transfer leading to long range electrostatic fields (in excess of the atomic potential due to the specimen). Note – reading further I see this possibility is covered in the manuscript in section 2.3.1.
- In the code blocks, many numerical terms are included without a leading zero, i.e. .5 or .1 rather than 0.5 and 0.1. The APA style guide recommends always including the leading zero for numbers than can exceed 1.0.
- In the show_atoms( … ) command, I wanted to tilt the cell to arbitrary angles to make sure I was constructing my sample correctly. It’s possible I am just missing something, but perhaps the argument “plane= arg” could be extended to allow arbitrary 3 element project vectors (u, v, w)?
- In the conclusion, the authors explicitly mention NION Swift’s python implementation. The other vendors are significantly behind on python implementation, but have made some progress which could also be mentioned (e.g. GMS python scripting tinyurl.com/kykj38bj).

Is the rationale for developing the new software tool clearly explained?

Yes

Is the description of the software tool technically sound?

Yes

Are sufficient details of the code, methods and analysis (if applicable) provided to allow replication of the software development and its use by others?

Yes

Is sufficient information provided to allow interpretation of the expected output datasets and any results generated using the tool?

Yes

Reviewer Expertise:

Transmission electron microscopy, electron scattering simulation, data analysis, materials science.

I confirm that I have read this submission and believe that I have an appropriate level of expertise to confirm that it is of an acceptable scientific standard.

# Response

**Published:** 2021-05-18

**Authors:** Toma Susi

Original comments by the reviewer are reproduced in *italics* below, followed by our point-by-point replies.

*In this manuscript, Madsen and Susi present their image simulation code abTEM for transmission electron microscopy. This code has many things to recommend it:*

- *completely open source*
- *all python implementation for easy understanding and modification*
- *GPU acceleration for fast calculations*
- *support for calculating the electrostatic atomic potentials from first principles (to include bonding, charge transfer, etc.) through the GPAW DFT code.*
- *support for atomic cell construction and manipulation via the ASE code.*
- *implementation of both classic algorithms such as multislice and newer methods like PRISM.*

* This paper is very well written, and the codes are well documented and user-friendly. I had a lot of trouble in windows, but the binder notebooks all appear to work well. I tested as many of the paper use cases as I could – all worked well, though it would be helpful for the authors to include more sample code for different sample geometries. I should note that I attempted to break the code by checking sampling densities and slice thicknesses that were far outside the range of ordinary simulation parameters (both too large and too small), and I was unable produce results that seemed unphysical – this shows the robustness of the potential integration pipeline developed in this code.*

*I also think the implementation of PRISM on the GPU is very clever – the batching of adjacent probes is a very clean way to efficiently use however much GPU RAM is available. It’s also pretty cool to see an all-python GPU implementation that is faster than Prismatic!*

*In summary, I recommend indexing of this work after minor revision. Below I have listed a few questions I have about the paper as well as minor comments that the authors are free to ignore.*

 We are very grateful for this very thorough and fair evaluation of our work! Below we address the points you raise in order.

*“ … allowing us to interpolate the measured images down to the sampling of the potential (0.04 Å) …” How is the interpolation performed? Fourier space padding I assume? If it’s real space, even bicubic interpolation may not be accurate enough.*

 Indeed, bicubic interpolation is not accurate enough, we used biquintic interpolation, which seems to be good enough in this case. Your comment prompted us to implement the option of doing the interpolation with Fourier space padding. We see that it does perform a bit better than biquintic interpolation, as you may have anticipated. We have a small example in our repository that demonstrates how well interpolation compares to a finer scan sampling:

https://github.com/jacobjma/abTEM/blob/master/examples/other/is_nyquist_enough.ipynb

*The images in Figure 2 look very strange to me. I checked the aspect ratio and it is correct (though it’s quite strange to label one axis in steps of 2.0 and the other in steps of 2.5 Angstroms). However, the atoms are definitely not round, which I assume is due an interpolation error. This can be seen if I saturate the image and draw a perfect circle.*

 Thank you for flagging these issues.  This kind of unequal adaptive tick spacing is unfortunately the default behavior in matplotlib. We have now implemented an “equal_ticks” argument for our show() methods, but leave this off by default since it will not play nicely with any scale updates. Perhaps a better solution can be found in the future, and we have updated the ticks of Figure 2 to have equal spacing.

 Regarding the non-round atoms: this seems to have been an issue with the cell we used for this example being slightly too small to fit the probe; we have now tiled the potential to avoid this and have updated the figure.

*I also don’t quite understand the results in Figure 2 d, e, f. The largest deviation in the BF signal is in the middle of the BN rings, furthest from the atomic potentials – I can rationalize this since this probe position is strongly affected by the degree of ionic screening. However, I would still like to see the quantitative intensity measurements, i.e. dimensions on the vertical axes of b, e, h in units the probe intensity*.

 That is indeed the case, but not only due to ionic screening: charge rearrangement due to bonding is most easily visibly at positions furthest away from the atoms, as the differences to the independent atom model are – in relative terms – the largest there. We also observed the same for graphene in our previous study (doi: 10.1016/j.ultramic.2018.11.002).  In the case of hBN, there is also a noticeable difference in the BF intensity of the N site, where in the case of the *ab initio* potential, the greater electronegativity of N in the bonded ionic compound leads to significant charge transfer from the B atoms, enhancing the screening of the N nucleus and thus lower its contrast (as originally shown in doi: 10.1038/nmat2941).

 We have now added scales to the line plot vertical axes to facilitate quantitative comparisons, which better show the relatively small magnitudes of the differences. We would add though that this is a simple example meant to illustrate how easy it is to use *ab initio* potentials; in a real study one would want to properly converge the DFT parameters and use a more realistic probe!

*Figure 5a caption or the paper text should probably mention the image is HAADF (I assume) with the detector annular ranges.*

 Indeed, this has been added.

*Figure 1 shows most of the important objects in abTEM. I would suggest adding a few more: the center-of-mass (along x and y direction) of the probe output intensity would make it easy to perform differential phase contrast (DPC) simulations (which might be already included as an output since DPC results are shown later in the paper)*. 

 The SegmentedDetector object already allows you to run DPC simulations using a circular detector. We also implement a center_of_mass function which allows you to calculate the center of mass and integrated COM from a 4D-STEM dataset. However, having a dedicated center-of-mass detector is a good idea, since writing the intermediate results of huge 4D-STEM datasets to disc may be bottleneck for computational performance. The center-of-mass detector will be added to abTEM in the near future.

*I would also like to see some kind of method for outputting a thickness series – it would be useful for researchers who wish to compare HRTEM images, single STEM probes, or PACBED images with experiments*. 

 In the example linked below, we show how performing a PACBED thickness series might be accomplished. While we believe the current implementation may be preferred by experienced Python users due to the amount of flexibility it allows, we also see that the current option is not very beginner-friendly. In a future release, we will aim to develop a simpler and more compact solution. https://github.com/jacobjma/abTEM/blob/master/examples/other/pacbed_thickness_series.ipynb

*Finally, it might be useful to include some kind of post-processing as a class, for example numerical integration over a range of angles or defocus values to model limited coherence (maybe this would belong in the Measurement class?)*.

 We uploaded an example showing how you might do this. Our above comment related to PACBED applies here as well, we will try to make the code for doing this more user-friendly. https://github.com/jacobjma/abTEM/blob/master/examples/other/partial_coherence_4DSTEM.ipynb

*Is the choice to go with MPI fixed? I might recommend Dask instead of (or in addition to) since it seems to be easier to scale from local parallelization to large scale HPC parallelization*.

 No, we actually reconsidered and decided to go with Dask shortly after the submission of the article. We have started development on this, but it is not ready to include in this version of the code, but we expect to include it in the next major release.

*“… is encoded in the scattering of the electron waves by the electrostatic potential of the specimen.” The electron wave can also scatter from electrostatic and magnetic fields too. I am particularly interested in using abTEM to simulate samples with significant charge transfer leading to long range electrostatic fields (in excess of the atomic potential due to the specimen). Note – reading further I see this possibility is covered in the manuscript in section 2.3.1*.

 We believe you are thinking in the context of the independent atom model, where electrostatic potentials are something additional to the atomic potentials. In terms of the physics, the electrostatic potential of the specimen naturally includes all contributions, atomic or charge transfer leading to long-range electrostatic fields, whose natural inclusion is one of the benefits of using an *ab initio* description!

*In the code blocks, many numerical terms are included without a leading zero, i.e. .5 or .1 rather than 0.5 and 0.1. The APA style guide recommends always including the leading zero for numbers than can exceed 1.0*.

 This has now been corrected.

*In the show_atoms( … ) command, I wanted to tilt the cell to arbitrary angles to make sure I was constructing my sample correctly. It’s possible I am just missing something, but perhaps the argument “plane= arg” could be extended to allow arbitrary 3 element project vectors (u, v, w)?*

 You can now set the plane normal using the azimuth and elevation angles in a tuple of length two, which will give you a 3D projection of the structure. However, since matplotlib does not have a real 3D engine, this implementation is a bit constrained. You may want to try ASE’s visualize.view() method, which opens an interactive 3D view. This also has some limitations, as it does not work seamlessly in remote environments and has trouble with large systems. In such cases you can use the nglview backend: with nglview installed you can specify “view(atoms, viewer=’’nglview)”. We have made nglview available in our Binder environment.

*In the conclusion, the authors explicitly mention NION Swift’s python implementation. The other vendors are significantly behind on python implementation, but have made some progress which could also be mentioned (e.g. GMS python scripting tinyurl.com/kykj38bj)*.

 That is a fair point, and GMS is now briefly mentioned in our conclusion
